# Venetoclax-based chemotherapy in acute and chronic myeloid neoplasms: literature survey and practice points

**DOI:** 10.1038/s41408-020-00388-x

**Published:** 2020-11-23

**Authors:** Naseema Gangat, Ayalew Tefferi

**Affiliations:** Division of Hematology, Mayo Clinic, Rochester, MN USA

**Keywords:** Acute myeloid leukaemia, Acute myeloid leukaemia, Myelodysplastic syndrome

## Abstract

Venetoclax (VEN), a small-molecule inhibitor of B cell leukemia/lymphoma-2, is now FDA approved (November 2018) for use in acute myeloid leukemia (AML), specific to newly diagnosed elderly or unfit patients, in combination with a hypomethylating agent (HMA; including azacitidine or decitabine) or low-dose cytarabine. A recent phase-3 study compared VEN combined with either azacitidine or placebo, in the aforementioned study population; the complete remission (CR) and CR with incomplete count recovery (CRi) rates were 28.3% and 66.4%, respectively, and an improvement in overall survival was also demonstrated. VEN-based chemotherapy has also shown activity in relapsed/refractory AML (CR/CRi rates of 33–46%), high-risk myelodysplastic syndromes (CR 39% in treatment naïve, 5–14% in HMA failure), and blast-phase myeloproliferative neoplasm (CR 25%); in all instances, an additional fraction of patients met less stringent criteria for overall response. Regardless, venetoclax-induced remissions were often short-lived (less than a year) but long enough to allow some patients transition to allogeneic stem cell transplant. Herein, we review the current literature on the use of VEN-based combination therapy in both acute and chronic myeloid malignancies and also provide an outline of procedures we follow at our institution for drug administration, monitoring of adverse events and dose adjustments.

## Introduction

Venetoclax (ABT-199) is a BH3-mimetic agent, a highly selective inhibitor of the anti-apoptotic protein B cell leukemia/lymphoma (BCL-2), which was discovered in 1984 as part of translocation t(14;18) in follicular lymphoma^1–3^. Since then several anti-apoptotic: BCLX_L_, MCL1, BCL-W and pro-apoptotic proteins: BAX, BAK, and BOK, and the BH3-only proteins BIM, BAD, BID, BIK, NOXA, and PUMA have been identified. Overexpression of BCL-2, BCLX_L_, and MCL1 frequently occurs in acute myeloid leukemia (AML) conferring resistance to conventional chemotherapy^4^. Initial clinical efforts with targeting anti-apoptotic proteins centered on navitoclax (ABT-263), a BH3 mimetic that binds to BCL-2, BCL-XL, and BCL-W^5^. As megakaryocytes are BCL-XL dependent, navitoclax caused significant dose-limiting thrombocytopenia limiting its utility in AML and other myeloid malignancies^6^. In additional preclinical work with ABT-737, an agent with similar activity to navitoclax, successful elimination of blasts in AML cell lines and patient samples was accompanied by eradication of BCL-2-dependent leukemia stem cells while sparing normal hematopoietic stem cells which rely on MCL1 for survival^7^. On the other hand, venetoclax, which is a modified BH3-mimetic derivative of navitoclax, maintains specificity for BCL-2 but lacks affinity for BCL-XL. Venitoclax also exhibited potent anti-leukemic activity in AML cell lines, patient samples, and xenograft murine models^8^. Interestingly, AML cell lines with MLL-fusion and samples from acute promyelocytic leukemia patients were particularly sensitive to venetoclax therapy^9^. Moreover, in preclinical models, synergy with the hypomethylating agent (HMA) azacitidine, which inhibits MCL1, was also established^10,11^. Together, these findings suggested promising activity of venetoclax in AML and laid the groundwork for clinical studies. In the current review, we summarize retrospective observations and clinical trials with venetoclax-based regimens in AML, including those that led to its FDA approval in November 2018 for treatment naïve elderly or unfit AML. Additionally, we share preliminary observations from ongoing studies in myelodysplastic syndromes (MDS) and related chronic myeloid malignancies. Furthermore, we expand upon practice relevant issues that are frequently encountered with use of venetoclax-based chemotherapy.

## Venetoclax as upfront therapy in AML

Venetoclax in combination with HMA or low-dose cytarabine has undergone extensive clinical evaluation in AML, with Phase 1b/II clinical trial data forming the basis of its FDA approval in November 2018 for treatment naïve, elderly >75 years old or unfit AML patients^12,13^. Please refer to Table 1 for a comprehensive summary of results from all published clinical trials and retrospective studies with venetoclax-based chemotherapy in treatment naïve AML. The initial Phase 1b study with venetoclax plus HMA in untreated AML comprised of three patient cohorts, namely venetoclax plus either (i) decitabine (Group A), (ii) azacitidine (Group B), or (iii) decitabine in addition to posaconazole (Group C), which established the recommended phase 2 dose of venetoclax at 400 or 800 mg daily^12^. In terms of responses, overall response rates were 62% with complete remission (CR) achieved in 27% with venetoclax plus azacitidine or 35% with decitabine^12^. In a subsequent phase 1b/II trial in AML patients above 65 years of age that were unfit for intensive chemotherapy, administration of venetoclax 400 or 800 mg with either azacitidine or decitabine resulted in a composite response (complete remission (CR, 37%)/CR with incomplete hematological recovery (CRi; 30%) rate of 67%)^13^. Notably, a rapid median time to first response at 1.2 months, with a median duration of response of 11.3 months, and superior median overall survival of 17.5 months were observed. Moreover, responses held across the spectrum of unfavorable cytogenetic and molecular genetic abnormalities such as *TP53, FLT3, IDH1/2* mutations. As expected, common adverse events included febrile neutropenia in 43% followed by myelosuppression in a quarter, and mild-moderate gastrointestinal toxicity. Early 30-day mortality was low (<5%). It should be emphasized that the study cohort comprised of a substantial proportion of patients harboring adverse cytogenetic anomalies (50%), with a quarter of patients with *TP53* or *IDH1/2* mutations and secondary AML. In order to elucidate the mechanism underlying response to venetoclax-based therapy, Pollyea et al.^14^ studied 33 patients treated with azacitidine plus venetoclax, and discovered treatment-induced disturbances in the tricarboxylic acid cycle with reduction in alpha ketoglutarate, increase in succinate levels along with inhibition of the electron transport chain, thereby eradicating leukemia stem cells^14^. The recently published VIALE-A phase-3 randomized study included elderly >75 years or younger AML patients if comorbidities precluded intensive therapy but was exclusive of patients with favorable cytogenetics (t(8;21, inv 16, t(15;17)), prior HMA exposure, or AML arising from myeloproliferative neoplasms (MPN). The study not only confirmed a superior response rate of 66% with combination of azactidine plus venetoclax vs 28% with azacitidine alone but also demonstrated an overall survival advantage of 5 months for venetoclax with azacitidine with a median overall survival of 14.7 months vs 9.6 months with azacitidine alone^15^. Moreover, superior responses occurred in *NPM1* (67% vs 24%), *FLT3* (72% vs 36%), *IDH1/2* (75% vs 11%), and *TP53* (55% vs 0%) mutated patients when treated with a combination of azacitidine and venetoclax as opposed to azacitidine alone^15^.

**Table 1:** Clinical studies with venetoclax-based chemotherapy in treatment naïve acute myeloid leukemia (AML).

Study	Design	Treatment arms	Toxicity	Efficacy	Survival	Correlative studies
DiNardo et al. *Lancet Oncol.* (2018)	Phase 1b Group A: *N* = 23 Group B: *N* = 22 Group C: *N* = 12	Group A (VEN + decitabine) Group B (VEN + azacitidine), Group C (VEN + decitabine+posaconazole) Dose escalation: Group A/B VEN 400 mg (cohort 1), 800 mg (cohorts 2/3), 1200 mg (cohort 4), 400 mg for group C >65 years unfit for induction therapy. ECOG 0-2, intermediate/poor cytogenetic risk	Febrile neutropenia Lung infection Nausea Vomiting Fatigue Leukopenia Thrombocytopenia Diarrhea Anorexia	RP2D 400 mg daily or 800 mg interrupted schedule. CR/CRi; 35 (61%) Median duration of response 8.4 months (group A), 12.3 months (group B), 4.3 months (group C)	Median OS; group A 15.2 months, group B 14.2 months	23/35 (66%) with intermediate-risk cytogenetics in CR/CRi vs 11/21 (52%) with poor-risk cytogenetics. Mutations and response: 10/17 (59%) with *IDH1/2* mutations, 3/4 (75%) with *FLT3-ITD* mutations; vs 4/11 (36%) with *TP53* mutations in CR/CRi
DiNardo et al. *Blood* (2019)	Phase 1b *N* = 145	Dose escalation, VEN 400, 800, or 1200 mg daily with decitabine or azacitidine. Dose expansion, 400 or 800 mg VEN with either HMA. >65 years unfit for induction therapy	Grade 3/4 AEs: febrile neutropenia (43%), leukopenia (31%), anemia (25%), thrombocytopenia (24%), pneumonia (13%)	CR/CRi 67% in all patients. CR/CRi 73% with VEN 400 mg. Median duration of CR/CRi (all patients)— 11.3 months	Median OS in all patients—17.5 months. Median OS (VEN 400 mg)—NR	CR/CRi with poor- and intermediate-risk cytogenetics 60% and 74%. CR/CRi *NPM1* mutation: 91.5%, *IDH1/2* mutations: 71%, *FLT3* mutation: 72%, *TP53* mutation: 47%
Wei et al. *JCO* (2019)	Phase 1b/II *N* = 82	VEN 600 mg po daily + LDAC 20 mg/m^2^ S/C (days 1–10) >60 years previously untreated AML ineligible for intensive chemotherapy	Grade 3/4 AEs: febrile neutropenia (42%), thrombocytopenia (38%), leukopenia (34%)	CR/CRi 54% Median duration of response; 8.1 months (95% CI, 5.3–14.9 months)	Median OS 10.1 months (95% CI, 5.7 to 14.2)	*NPM1*, *IDH1/2* mutations higher CR/CRi (89%/72%, respectively), *TP53* or *FLT3* mutations lower CR/CRi (30%/44%, respectively).
DiNardo et al. *NEJM* (2020)	Phase-3 placebo-controlled, randomized, VIALE-A *N* = 433	Azacitidine + VEN (*N* = 286) or azacitidine + placebo (*N* = 145). 2:1 ratio >75 years or >18 years with comorbidity ineligible for intensive induction therapy	Nausea, constipation, diarrhea, vomiting. SAEs febrile neutropenia (30% vs 10%), pneumonia (17% vs 22%) with and without VEN, respectively. 1% tumor lysis with VEN	CR + CRi 66.4 vs 28.3% (*p* < 0.001) with and without VEN, respectively	Median OS 14.7 and 9.6 months in the VEN vs placebo arm, respectively (HR, 0.66; 95% CI, 0.52–0.85; *p* < 0.001)	CR/CRi rate: *IDH1/2* (75% vs 11%), *FLT3* (72 vs 36%), *NPM1* (67% vs 24%), and *TP53* (55% vs 0%), with and without VEN respectively.
Wei et al. *Blood* (2020)	Phase-3 randomized double-blind placebo-controlled trial *N* = 211	VEN (*n* = 143) or placebo (*n* = 68) in 28-day cycles, + LDAC days 1–10, 2:1 ratio. ≥18 years newly diagnosed AML ineligible for intensive chemotherapy	Grade 3/4 AEs: (VEN vs LDAC alone) febrile neutropenia (32% vs 29%), neutropenia (47% vs 16%), thrombocytopenia (45% vs 37%)	CR/CRi 48%/13% for VEN + LDAC vs LDAC alone	Median OS 7.2 (VEN + LDAC) vs 4.1 (LDAC) months (*p* = 0.11). Additional 6-month follow-up median OS 8.4 months (VEN + LDAC) (*p* = 0.04)	Higher CR/CRi with *TP53* mutation (18% vs 0%), *IDH1/2* (57% vs 33%), *NPM1* (78% vs 57%) with VEN + LDAC vs LDAC + placebo. No difference with *FLT3* mutation (44% vs 45%) with VEN + LDAC vs LDAC + placebo
Winters et al. *Blood Adv.* (2019)	Retrospective study *N* = 33 treated off-trial compared to 33 pts treated on trial	VEN 400 mg daily, for 28-day cycles. AZA 75 mg/m^2^ IV or S/C days 1–7	Neutropenia Anemia Thrombocytopenia Neutropenic fever Pneumonia Fatigue	CR/CRi 63.3% vs 84.9% for off-trial and on trial patients, respectively (*p* = 0.081)	Median OS for off-trial patients 381 days vs 880 days for trial patients (*p* = 0.041)	CR/CRi rates lower with prior HMA. Off-trial patients without prior HMA; 19/26 (73.1%) vs with prior HMA; 0/4 (0%)
Morsia et al. *AJH* (2020)	Retrospective study *N* = 44 compared to 56 elderly pts treated with HMA alone	Median dose of VEN 150 mg (50–400 mg). AZA 75 mg/m^2^ IV or S/C days 1–7 or decitabine 20 mg/m^2^ IV days 1–5	Infectious complications in 17/44 (38%), heart failure 5/44 (11.4%), bleeding 4/44 (9.1%), tumor lysis 2/44 (4.5%), and renal failure 2/44 patients (4.5%)	CR/CRi (56.4%) with VEN/HMA, vs 23% with HMA alone (*p* = 0.005).	Median OS 17 months vs 3 months with/without CR/CRi, *p* = 0.0009)	4/4 (100%) with *CEBPA* mutation achieved CR/CRi vs 18/35 (51%) in *CEPBA* wild type

*VEN* venetoclax, *ORR* overall response rate, *CR* complete remission, *CRi* complete remission with incomplete hematological recovery, *RP2D* recommended phase 2 dose, *OS* overall survival, *HMA* hypomethylating agent, *NR* not reached, *AE* adverse event, *SAE* serious adverse event, *HR* hazard ratio, *CI* confidence interval, *LDAC* low-dose ara-C, *AZA* azacitidine.

Dinardo et al.^16^ shared phase 2 results with a 10-day course of decitabine 20 mg/m^2^ with venetoclax 400 mg daily for induction followed by 5 days of decitabine with venetoclax in consolidation in 70 treatment-naïve elderly AML patients over 60 years that were ineligible for intensive therapy. Unsurprisingly, decitabine dose reductions were instituted in 13% with over 90% of patients receiving 21 days or less of venetoclax with infectious complications recorded in half of patients. CR/CRi was achieved in 84%, with minimal residual disease (MRD) negativity in 67%, with high responses across all ELN-risk groups; CR/CRi rates of 90%, 100%, and 75% among favorable, intermediate, and adverse risk groups, respectively^16^. Similarly, CR/CRi rates among *NPM1*, *IDH1/2*, *N/Kras*, and *TP53*-mutated patients were 95%, 84%, 74%, and 69%, respectively. Importantly, responses were less durable and similar with a 5- vs 10-day course of decitabine plus venetoclax in *TP53* mutated patients. In contrast, for all treatment-naïve patients median duration of response was not reached, with a median overall survival of 18.1 months^16^. Interestingly, among 14 newly diagnosed *FLT3*-mutated patients, ten received FLT3 inhibitors in addition to decitabine and venetoclax, achieving CR/CRi rates and MRD negativity by PCR in 86% with three patients transitioning to allogeneic hematopoietic stem cell transplant (AHSCT)^16^.

Venetoclax is also administered in combination with low-dose cytarabine in AML, particularly in patients experiencing disease progression on HMA therapy^17,18^. This regimen was studied in a phase 1b/II trial in which venetoclax 600 mg orally daily was administered in combination with low-dose cytarabine 20 mg/m^2^ subcutaneously days 1–10 to elderly AML patients, half with secondary AML, and one-third each with poor-risk cytogenetics or prior HMA exposure^17^. Among 82 elderly AML patients (median age: 74 years) treated on study, CR/CRi rates were 54% with CR rate of 21%. As expected, higher responses were noted with de novo AML, intermediate-risk cytogenetics, and in the absence of prior HMA exposure. Once again, median time to CR/CRi was rapid at 1.4 months with median duration of response of 8 months. Median overall survival for the entire cohort was 10.1 months, with distinct survival differences appreciated based on prior HMA use (4.1 vs 13.5 months, respectively, with or without prior HMA). The follow-up randomized phase-3 study, VIALE-C which included AML patients >18 years of age ineligible for intensive therapy, also confirmed superior CR/CRi rate at 48% with venetoclax plus low-dose cytarabine compared to 13% with cytarabine alone^18^. Upon initial survival analysis, an overall survival benefit with the combination of venetoclax and low-dose cytarabine was not apparent; however, with an additional 6-month follow-up, survival distinctions emerged with a median overall survival of 8.4 months for venetoclax plus cytarabine vs 4.1 months with cytarabine alone. Superior responses were recorded with combination therapy among *NPM1* (78% vs 57%), *IDH1/2* (57% vs 33%), and *TP53* (18% vs 0%) mutated patients with no difference noted in *FLT3*-mutated patients (44% vs 45%).

Moving beyond clinical trials which are fraught with issues of selection bias and vigilant monitoring, Winters et al. shared their real-world experience with azacitidine plus venetoclax in AML. Thirty-three patients treated with azacitidine plus venetoclax off-trial (*n* = 33) at their institution were compared with trial patients treated with the same regimen. Not surprisingly, lower response rates (63% vs 85%, *p* = 0.08) with consequently shortened survival (381 vs 880 days, *p* = 0.04) were noted among off-trial patients^19^. Moreover, none of four patients with prior HMA exposure responded with 19 of 26 (73.1%) patients without prior HMA responding to therapy. On that note, we have recently published our Mayo clinic experience with off-trial use of HMA plus venetoclax among 44 treatment-naïve AML patients of median age 73.5 years, which were enriched with secondary, therapy related and ELN adverse risk disease. We found encouraging responses with CR/CRi rate of 50%, albeit lower than that of clinical trial reports^20^. Remarkably, one-third of our patients achieved response after three or fewer cycles of therapy and four patients (9.1%) proceeded to AHSCT. Prior HMA exposure did not impact response outcome with three of five such patients achieving CR/CRi. Another noteworthy observation from our study was the association of *CEPBA* biallelic mutations with a favorable response, with all four patients harboring *CEPBA* biallelic mutations responding to therapy vs 18 of 35 (51%) *CEBPA* wild-type patients. Even though CR/CRi rates in our series were superior with HMA plus venetoclax in comparison to a historical cohort of elderly AML patients treated with HMA alone (50% vs 23%), a substantial improvement in median overall survival was not detected; median overall survival of 11 months with HMA plus venetoclax vs 9.5 months with HMA alone^20,21^.

## Venetoclax as salvage therapy in AML

In the foremost Phase II study of venetoclax in AML, the drug was administered as monotherapy at 800 mg daily to 30 patients with relapsed/refractory disease, exhibiting fairly limited activity with an overall response rate of 19%^22^. However, it was striking that one-third of patients in CR/CRi, harbored *IDH1/2* mutations, consistent with later reports of venetoclax combination therapy in treatment-naïve AML reaffirming the sensitivity of *IDH1/2* mutated patients to venetoclax-based therapy^22–24^. A recent phase II study with a 10-day induction course of decitabine along with venetoclax in 55 relapsed/refractory AML patients of which one-third were relapsed post AHSCT showed reasonable efficacy^16^. Remarkably, CR/CRi rates were 42% with half of patients achieving MRD negativity with favorable responses in *NPM1, IDH1/2*, and *FLT3* mutated patients^16^. Moreover, median duration of response was 16.8 months with median overall survival of 7.8 months. Among 12 patients with previously treated FLT3 mutated AML, 8 with prior FLT3 inhibitor exposure, all received FLT3 inhibitors along with decitabine and venetoclax achieving a CR/CRi rate of 42%, with MRD negativity by flow cytometry and PCR in half and quarter of responding patients respectively. Furthermore, four patients proceeded to AHSCT^16^.

Since approval of venetoclax in 2018 for upfront use in AML, it has gained popularity as a salvage regimen with a handful of published retrospective reports that are delineated in Table 2 (refs. ^20,25–34^). We recommend exercising caution while interpreting findings from these reports due to immense heterogeneity in patient population studied (inclusion of relapsed MDS, other myeloid malignancies, prior HMA exposure, post AHSCT), in addition to variations in dose and schedule of treatment regimens utilized either as monotherapy, combination with HMA or low-dose cytarabine. As a result, reported responses with venetoclax-based regimens in the relapsed AML setting are highly variable. For instance in an MD Anderson series (*n* = 43) which also included two patients each with MDS and blastic plasmacytoid dendritic cell neoplasm, 72% received HMA and the remainder low-dose cytarabine, with overall response rate of 21% comprising of two patients in CR, three in CRi, and four in morphological leukemia free state (MLFS)^25^. In contrast, a study from City of Hope (*n* = 33), in which relapsed AML patients were treated with venetoclax in conjunction with HMA (decitabine, 5-day or 10- day course (*n* = 15 and 16, respectively), azacitidine (*n* = 2), an overall response rate of 64% with ten patients in CR, seven in CRi, and four in MLFS were reported^26^. These two studies also differed in regard to predictors of response with high response rate of 50% in *RUNX1-*mutated patients in the MD Anderson series^25^; on the other hand 44% of *FLT3-*mutated patients responded in the latter study^26^. However, both studies demonstrated similar responses among *TP53*-mutated patients of 50% and 67%, respectively. A follow-up updated analysis of 90 relapsed AML patients treated at City of Hope, half with prior HMA use, and a third relapsed post-transplant yielded CR/CRi rates of 46%, with *TET2* and *ASXL1* mutations associated with an improved response^34^. Moreover, one-third of responders from this study proceeded to AHSCT^34^.

**Table 2:** Clinical studies with venetoclax-based chemotherapy in relapsed/refractory acute myeloid leukemia (AML).

Study	Design	Treatment arms	Toxicity	Efficacy	Survival	Correlative studies
Konopleva et al. *Cancer Discov.* (2016)	Phase II *N* = 32	Single arm VEN 800 mg daily. RR AML (*n* = 30) or unfit for intensive therapy (*n* = 2)	Garde3/4 AE Nausea Vomiting diarrhea Febrile neutropenia Hypokalemia	Response by revised IWG criteria ORR: 19% CR: 6% CRi: 13% PR: 19% Median duration of CR: 48 days	Median LFS: 2.3 months Median OS: 4.7 months	4/12 (33%) with *IDH1/2* mutations achieved CR/CRi
DiNardo et al *AJH* (2018)	Retrospective *N* = 43	VEN + HMA therapy (*n* = 31, 72%); eight (19%) received (LDAC) R/R AML (*n* = 39), MDS (*n* = 2), BPDCN (*n* = 2).	31 (72%) with grade ≥ 3 infection,	ORR in 9 (21%) patients, 2 CR, 3 CRi, 4 MLFS.	Median survival 3.0 months (range, 0.5-8.0),	Responses in 5/21 (24%) with intermediate-risk cytogenetics, 3/11 (27%) *IDH1/2* mutant, 4/8 (50%) *RUNX1* mutated patients. 2/10 (50%) *TP53* mutated responded both with concurrent *RUNX1* mutation. Objective response achieved in 3/20 (15%) with adverse cytogenetics, all with *RUNX1* mutation.
Aldoss et al. *Haematologica* (2018)	Retrospective *N* = 33	VEN 400 mg daily (200 mg daily if on azole). Decitabine 20 mg/m^2^ × 5 days (*n* = 15) or 10 days (*n* = 16). 5-azacitidine (*n* = 2) 75 mg/m^2^ for 7 days	SAE sepsis (*n* = 11), pneumonia (*n* = 5), colitis and diarrhea (*n* = 3), atrial fibrillation (n = 2), acute renal failure (*n* = 2)	ORR 64% (N = 21); 10 (30%) patients achieved CR, 7 (21%) CRi and 4 (12%) MLFS	1- year OS for all patients 53%	Response rate for molecular mutations: 67% for *IDH 1/2* mutation, 44% for *FLT3* mutation (ITD or TKD), and 67% with *TP53* mutation
*Aldoss* et al. AJH (2019)	Retrospective *N* = 90	VEN + Decitabine (*n* = 81) Cycle 1 decitabine 10 days (*n* = 48) VEN + azacitidine (*n* = 9) RR AML	Not available	CR/CRi 46% (*n* = 41) CR *n* = 23 (26%) CRi *n* = 18 (20%)	Median OS for all patients 7.8 months, 16.6 months for patients in CR/CRi vs 5.1 months for patients who did not respond	ELN genetic risk associated with reduced CR/CRi, *ASXL1* and *TET2* mutations associated with better CR/CRi
Ram et al. *Ann Hematol* (2019)	Retrospective *N* = 23	VEN 400 mg with (azacitidine, *n* = 16, decitabine, *n* = 4) or LDAC (*n* = 3) AML patients relapsed/refractory to HMA	Febrile neutropenia (78%)	CR/CRi (43%) (*n* = 10)	OS 74% at 6 months, Median OS 5.6 months, median OS with CR/CRi 10.8 months	Blast % in bone marrow/peripheral blood inversely correlated with CR
Gaut et al. *Leuk. Res.* (2020)	Retrospective *N* = 14	VEN + azacitidine (*n* = 8), decitabine (*n* = 5), LDAC (*n* = 1). RR AML	Grade 3/4 Infection (*n* = 7, 50%), intracranial hemorrhage (*n* = 3, 21.4%).	ORR, 35.7%. CR/CRi (*n* = 3) PR (*n* = 2)	Median OS 4.7 months	*NPM1* mutated patient achieved CRi, No response in *IDH2* mutated patient, 2/4 responses in *RUNX1* mutated. 3/44 *FLT3* mutated and/4 *TP53* mutated had response
Wang et al *Ann. Hematol.* (2020)	Retrospective *N* = 40	VEN monotherapy (*n* = 8), VEN + azacitdine (*n* = 21) + LDAC (*n* = 10), or FLAG (*n* = 1). RR AML	Neutropenic fever 67.5% (*n* = 27), 45% (*n* = 18) with documented infections.	ORR 50% CR (*n* = 5), CRi (*n* = 4) MLFS (*n* = 5) PR (*n* = 6).	Median OS 6.6 months.	ORR 100% *FLT3*-TKD (*n* = 2), *SRSF2* (*n* = 5), *NPM1* (*n* = 3), *U2AF1* (*n* = 2) mutations, 77.8% for *ASXL1* mutations (*n* = 9), 75% *IDH2* (*n* = 4), or *STAG2* mutations (*n* = 4), 54.5% for *RUNX1* mutations
Huemer et al. *Eur. J. Haematol.* (2019)	Retrospective N = 7	VEN monotherapy 800 mg daily secondary AML refractory to HMA	Not available	CR 2/7 (28.6%)	Median OS from VEN initiation 55 days (15–549 days)	High BCL‐2 and/or BIM expression in myeloblasts found in responders
Ganzel et al. *Leuk. Lymphoma* (2020)	Retrospective *N* = 40	VEN + HMA (62.5%). VEN + LDAC(22.5%). VEN monotherapy (15%). RR AML	Gastrointestinal (*n* = 4), infections (including 1 pulmonary aspergillosis) (*n* = 3), skin complications, (*n* = 2), weakness (*n* = 2), vertigo (*n* = 1)	CR/CRi 37.5% (*n* = 15)	Median OS from VEN initiation 5.5 months	Not available
Byrne et al. *AJH* (2020)	Retrospective *N* = 21	VEN + HMA (*n* = 16) VEN + LDAC (*n* = 5) Relapsed AML s/p allogeneic transplant for myeloid disease	Infectious complications (*n* = 13)	ORR 8/19 (42.1%) CR (*n* = 5) CRi (*n* = 3)	Median OS 7.8 months.	None of the 4 patients with complex karyotype and *TP53* mutation responded
Morsia et al. *AJH* (2020)	Retrospective *N* = 42	VEN + HMA RR AML excluding post-transplant relapse	Infectious complications in 85.7% (*n* = 36), heart failure in 19% (*n* = 8), renal failure 4.8% (*n* = 2) in the absence of tumor lysis	CR/CRi 14/42(33.3%) CR 19% (*n* = 8) CRi 14.3% (*n* = 6) PR 7.1% (*n* = 3)	Median OS 5 months (95% CI, 3–9 months); 15 months for those in CR/CRi vs 3 months for those not in CR/CRi	CR/CRi in *FLT3* (50%), *IDH2* (60%), *RUNX1* (75%), and *TP53* (40%) mutated patients (*p* = 0.77). Superior responses with *DNMT3A* (*n* = 4), *BCOR* mutations (*n* = 2) with all in CR/CRi.
Joshi et al. *BCJ* (2020)^59^	Retrospective *N* = 29	VEN + HMA (*n* = 26) VEN (*n* = 1) VEN + LDAC (*n* = 1) VEN + gilteritinib (*n* = 1) Relapsed AML (*n* = 19) or high grade MDS (*n* = 10) s/p allogeneic transplant	Grade 3/4 Neutropenia (*n* = 20) Thrombocytopenia (*n* = 19) Infections (*n* = 16) Anemia (*n* = 15)	ORR 38% (*n* = 11) CR/CRi 28% (*n* = 8) PR 10% (*n* = 3)	Median OS: 79 days, responders vs non-responders 403 days vs 55 days	Not available

*VEN* venetoclax, *RR* relapsed/refractory, *AE* adverse event, *IWG* international working group, *ORR* overall response rate, *CR* complete remission, *CRi* complete remission with incomplete hematological recovery, *PR* partial remission, *LFS* leukemia-free survival, *OS* overall survival, *RP2D* recommended phase 2 dose, *HMA* hypomethylating agent, *LDAC* low-dose ara C, *MDS* myelodysplastic syndrome, *BPDCN* Blastic plasmacytoid dendritic cell neoplasm, *MLFS* morphological leukemia free state, *SAE* serious adverse event, *CI* confidence interval.

In our Mayo clinic experience with venetoclax plus HMA as salvage therapy in relapsed AML exclusive of post-transplant relapse (*n* = 42), we observed CR/CRi rates of 33%, with similar responses across the mutational spectrum; *FLT3* (50%), *IDH2* (60%), *RUNX1* (75%), and *TP53* (40%) mutated patients. Furthermore, 8 of 42 patients (19.1%) were successfully bridged to AHSCT^20^. Similarly, a recent meta-analysis which included 224 patients with relapsed AML treated with venetoclax monotherapy or combination therapies demonstrated an overall response rate of 34.7%^35^.

In regard to venetoclax use as salvage therapy in post AHSCT patients, a recent review of 21 patients solely focused on relapsed AML following transplant performed for either AML (*n* = 16) or chronic myeloid malignancy (*n* = 2, MDS, *n* = 1 each with chronic myelomonocytic leukemia (CMML) and primary myelofibrosis)^32^. The majority of patients received venetoclax with HMA (*n* = 16). Eight of 19 evaluable patients (42%) responded with CR (*n* = 5) and CRi (*n* = 3). It is to be noted that half of these patients had relapsed within 6 months of transplant with a quarter within 100 days attesting to a population with dismal outcomes, despite which responses were sustained beyond 3 months in most patients with only one patient in CR/CRi progressing after 9 months of therapy. Moreover, four of eight patients were subsequently salvaged with either donor lymphocyte infusion (*n* = 2) or a second transplant (*n* = 2). Contrary to favorable responses reported in *TP53* mutated patients^36^, all four patients with complex karyotype and *TP53* mutation did not respond to therapy.

## Venetoclax in MDS and other chronic myeloid malignancies

Venetoclax is being investigated in clinical trials in both treatment naïve high-risk MDS and MDS with progression on HMA. Table 3 provides early observations from ongoing studies in MDS. A phase 1b study in treatment naïve MDS includes patients with intermediate 2 or high International Prognostic Scoring System (IPSS) risk categories or intermediate, high or very high Revised IPSS (IPSS-R) categories with <20% bone marrow blasts^37^. Of note, the above study excluded therapy-related MDS, myelodysplastic/myeloproliferative (MDS/MPN) overlap entities, and prior chemotherapy or AHSCT. Updated results were presented at the American society of hematology (ASH) annual meeting in 2019; a total of 59 treatment naïve MDS patients were treated with azacitidine and venetoclax on the dose escalation phase (100, 200, 400 mg venetoclax daily, *n* = 25) establishing 400 mg daily as the recommended dose for 14 days of a 28-day cycle followed by an expansion cohort (*n* = 22). Unlike AML, no dose ramp up was performed. Common toxicities consisted of myelosuppression and gastrointestinal symptoms, with febrile neutropenia in one-third of patients. Of the 57 patients evaluated for response, median time to response was 1 month (range: 0.7–3.5 months) with 18 patients (32%) achieving CR, 22 patients (39%) in marrow CR (mCR), and hematological improvement in 28 patients. Despite short follow-up, ten patients proceeded to AHSCT and an 18-month survival estimate for the cohort was 74%. Furthermore, when responses were correlated with IPSS-R cytogenetic categories, CR/mCR was achieved in almost all patients in the very good (100%) and good (94%) risk groups vs 62% and 73% in the poor and very poor-risk categories, respectively^38^.

**Table 3:** Clinical studies with venetoclax-based chemotherapy in myelodysplastic syndromes (MDS) and other myeloid malignancies.

Study	Design	Treatment arms	Toxicity	Efficacy	Survival	Correlative studies
Wei et al. *Abstract* *ASH* (2019), Garcia et al. *Abstract EHA* (2020)	Phase 1b *N* = 59	VEN oral for 14 days of each 28-day cycle, with cohorts 100-400 mg daily. Aza 75 mg/m^2^ days 1 to 7. Treatment naïve higher risk MDS.	Anemia, neutropenia including febrile neutropenia, thrombocytopenia. Constipation, nausea, diarrhea, and vomiting	MTD-400 mg ORR: CR 22/57 (39%) Stable disease 11/57 (19%) HI 28/56 (50%)	18-month estimate OS 74%	CR + mCR by IPSS-R cytogenetics: Very good 100% (2/2) Good 94% (17/18) Intermediate 63% (5/8) Poor 62% (8/13) Very poor 73% (11/15)
Zeidan et al. *Abstract ASH* (2019)	Phase 1 b *N* = 46	Cohort 1 (*N* = 22) VEN monotherapy, 400 mg (Arm A) or 800 mg (Arm B) per cycle (28 days). Cohort 2 (*N* = 24) Aza+ escalating doses of VEN: 100, 200, and 400 mg daily for 14 of 28-day cycles. Aza at 75 mg/m^2^ for the first 7 days of each cycle. Failure of HMA after at least 4 cycles of Aza or decitabine within 5 years	Grade 3 and 4 TEAEs Neutropenia (41%), thrombocytopenia (30%), leukopenia (24%), and anemia (15%)	Cohort 1: ORR 7% (1/16). Stable disease 75% (12/16). Cohort 2: ORR 50% (12/24). 13% (3/24) in CR 38% (9/24) in mCR. Stable disease 41% (10/24)	Cohort 1: Median PFS 3.4 months 6-month estimate OS 57% Cohort 2: Median PFS, OS not reached. 6-month estimate PFS 76% Estimate OS at 9-month 83%	Not available.
Zeidan et al. *Abstract* *EHA* (2020)	Phase 1b update *N* = 38	VEN escalation doses: 100, 200 and 400 mg daily for 14 of 28-day cycles. Aza at 75 mg/m^2^ for the first 7 days of each cycle. RR MDS Excluded prior AHSCT	Grade 3/4 AE Neutropenia (50%), thrombocytopenia (42%), leukopenia (39%), febrile neutropenia (29%), anemia (16%). Pneumonia (16%)	CR+ mCR (*n* = 15) 3 CR 12 mCR HI 25% mCR+HI 42%	Median PFS 9.1 months. 12-month OS 65%	Not available
Ball et al. *Blood Adv.* (2020)	Retrospective *N* = 44	VEN + HMA 61% azacitidine 75 mg/m^2^ × 7 days and 39% decitabine 20 mg/m^2^ × 5 days. Treatment naïve and HMA failure MDS (73%)	AE with discontinuation in 20%, anemia (*n* = 1), thrombocytopenia (*n* = 1), neutropenia (*n* = 2), neutropenic fever (*n* = 4), and unknown (*n* = 1).	ORR 59%, 14% CR, 27% mCR with HI, 18% mCR without HI	Median 0 S 19.5 months for all patients, 11.4 months among patients with HMA failure	Univariate analysis, very poor-risk IPSS-R cytogenetics associated with a significant decrease in overall response. HMA exposure did not affect response
Azizi et al. *Leuk. Lymphoma* (2020)	Retrospective *N* = 20	VEN + HMA Azacitidine (*n* = 8) Decitabine (*n* = 12) Treatment naïve and HMA failure MDS	Neutropenic fever (35%)	ORR (75%) CR (5%) mCR (60%) PR (10%)		Similar response with *RUNX1, TP53, TET2, ASXL1* mutations
Cortes et al. *Abstract ASH* (2019)	Retrospective *N* = 12	VEN 200 mg (range, 100–400 mg) with median duration of 14 days (range, 7–21). VEN + HMA (66%), VEN + LDAC (8.5%), VEN + LDAC + Cladribine+ Mylotarg (8.5%), VEN + LDAC + Cladribine+ Ruxolitinib (8.5%), VEN + CPX-351+Mylotarg (8.5%). MDS (*n* = 9) CMML (*n* = 3) HMA failure (*n* = 10)	11 patients (92%) with infections and 3 (25%) died from septic shock.	ORR 55% (*n* = 5) CR 11% (*n* = 1) mCR 22% (*n* = 2) HI 22% (*n* = 2)	Median OS 8.6 months (95% CI, 1.8–15.4 months)	Not available
Gangat et al. *BJH* (2020) In press	Retrospective *N* = 12	VEN + HMA Blast phase MPN	Not available	ORR 5/12 (42%) CR 3/12 (25%) PR 2/12 (17%)	Not available	CR rate higher with VEN + HMA (25%) compared to HMA alone (4%; *p* = 0.048) but both inferior to intensive chemotherapy (35%; *p* < 0.0001)

*VEN* venetoclax, *Aza* azacitidine, *MTD* maximum tolerated dose, *ORR* overall response rate, *CR* complete remission, *HI* hematological improvement, *IPSS-R* Revised International Prognostic scoring system, *HMA* hypomethylating agent, *TEAE* treatment emergent adverse event, *mCR* marrow CR, *PFS* progression free survival, *OS* overall survival, *RR* relapsed/refractory, *AHSCT* allogeneic hematopoietic stem cell transplant, *AE* adverse event, *PR* partial remission, *LDAC* low-dose ara-C, *CMML* chronic myelomonocytic leukemia, *MPN* myeloproliferative neoplasm.

Venetoclax-based therapy is also under investigation in MDS with disease progression after four cycles of HMA, administered as monotherapy in 22 patients (400 mg vs 800 mg per 28-day cycle) and in conjunction with azacitidine with escalating doses of venetoclax (100, 200, and 400 mg for 14 days) in 24 patients^39^. With monotherapy, overall responses were low occurring in only one of sixteen patients with stable disease in the majority (12 of 16 patients). Improved overall responses were recorded in half of patients treated with combination therapy, with 13% and 38% achieving CR, and mCR, respectively. Moreover four patients proceeded to AHSCT. Updated results of 37 evaluable patients with relapsed MDS excluding CMML and post AHSCT, treatment with combination therapy resulted in CR (*n* = 3) plus mCR (*n* = 12) in a total of 15 patients (40%) with median time to response of 1.2 months. An additional 25% of patients experienced hematological improvement with red cell or platelet transfusion independence noted in one-third of patients. An encouraging median progression free survival of 9 months with a 1 year overall survival estimate of 65% was also reported^40^.

A recent multi-institutional retrospective analysis of forty-four treatment naïve or relapsed MDS patients (IPSS-R very high risk (41%), poor or very poor-risk cytogenetics (43%), therapy-related MDS (34%), prior treatment with HMA (73%), and >10% marrow blasts (57%)) treated with a combination of venetoclax with either azacitidine or decitabine, yielded an overall response rate of 59% with two-thirds of patients successfully bridged to AHSCT^41^. Response breakdown was as follows: 14% CR, 27% mCR with hematologic improvement, and 18% mCR without hematological improvement. The vast majority of patients (79%) received either 400 or 200 mg venetoclax which was administered for 28 days in 77% of cases. Of note, one-fifth of patients discontinued therapy due to myelosuppression. Patients with very poor-risk IPSS-R cytogenetic abnormalities were less likely to respond with an overall response of 30% whereas prior HMA exposure did not impact response.

Two additional retrospective studies by Azizi et al (*n* = 20) and Cortes et al. (*n* = 12) that included both treatment naïve MDS and those with HMA failure reported CR plus mCR rate of 65% and 33%. respectively^42,43^. In the latter study which was inclusive of three patients with CMML and ten patients with HMA failure, venetoclax was administered in combination with HMA in two-thirds of the patients with the remainder receiving various combinations of venetoclax with low-dose cytarabine, cladribine, mylotarg, CPX-351, or ruxolinitib.

In terms of other chronic myeloid malignancies, limited data exist on venetoclax use in MPN and MDS/MPN overlap syndromes such as CMML. In myelofibrosis, clinical efforts are currently focused on a related drug, navitoclax, in combination with ruxolitinib^44^. We recently reported outcomes with venetoclax and HMA combination therapy in 12 patients with blast-phase MPN, with an overall response rate of 42%, comprising of three patients achieving CR (25%) and two with partial response (PR). Impressively, three of five responding patients transitioned to AHSCT^45^. In comparison to historical controls from the Mayo Clinic database of patients with blast-phase-MPN treated with HMA alone (*n* = 26) or intensive chemotherapy (*n* = 69), CR rate of 25% with HMA plus venetoclax was higher compared to those receiving HMA alone (4%; *p* = 0.048) and both were inferior to those receiving intensive chemotherapy (35%; *p* < 0.0001); furthermore, an additional 24% of patients receiving intensive chemotherapy achieved CRi but none among patients receiving HMA alone or HMA with venetoclax^46^.

## Practice points with venetoclax use

Venetoclax at 400 mg orally daily is administered most frequently in combination with hypomethylating agents (azacitidine 75 mg/m^2^ subcutaneously or intravenous × 7 days or decitabine 20 mg/m^2^ intravenously days 1–5 every 28 days). The combination of venetoclax 600 mg orally daily with low-dose cytarabine subcutaneously at 20 mg/m^2^ SC days 1–10 every 28 days is considered in patients with prior HMA exposure. On the other hand, venetoclax monotherapy is rarely used due to its limited activity. Concomitant use of medications that inhibit CYP3A4 and P-glycoprotein, particularly azole antifungals (i.e. fluconazole, voriconazole, and posaconazole) mandate venetoclax dose adjustments^47^. In other words, venetoclax dose with fluconazole is 200 mg (half dose) and with voriconazole or posaconazole is 100 mg daily (quarter dose). At our institution, we routinely prescribe antimicrobial prophylaxis for AML patients with acyclovir and levofloxacin in addition to azole antifungal prophylaxis^48^. Fluconazole is preferred in treatment naïve elderly patients while posaconazole is used in treatment naïve young AML patients or in the relapsed setting. Duration of venetoclax therapy is typically 28 days for cycle 1 in AML vs 14 days for MDS.

Newly diagnosed AML patients are hospitalized for venetoclax dose ramp up over 3 days (100, 200, and 400 mg without azoles, 50, 100, and 200 with fluconazole, or 20, 50, and 100 mg with posaconazole). Tumor lysis prophylaxis with saline hydration and allopurinol is instituted upon admission. We consider febuxostat in instances of renal dysfunction^49^ while rasburicase 6 mg IV fixed dose is reserved for high-risk patients presenting with hyperleukocytosis and markedly elevated uric acid^50^. In patients presenting with leukocytosis >25 × 10^9^/L, cytoreduction with hydroxyurea is promptly initiated with consideration of leukapharesis if symptomatic hyperleukostasis. We administer venetoclax once leukocyte count is below 25 × 10^9^/L. As a result, tumor lysis is extremely infrequent in our practice.

Following venetoclax ramp up and completion of HMA therapy, if medically stable, patients are discharged for daily outpatient monitoring with supportive care at our hospital based outpatient unit. The routine use of growth factor support is not encouraged at our institution; however, it is reasonable to consider in case of a non-resolving infection, or prolonged myelosuppression, if MLFS or CRi is achieved. A bone marrow biopsy is performed for response assessment after completion of cycle 1 (day 28) regardless of peripheral blood counts^51^. Once CR is achieved after cycle 1, we proceed with cycle 2 of therapy without dose modifications if platelets >100 × 10^9^/L and ANC > 1.0 × 10^9^/L. In the event of CRi post cycle 1, we interrupt therapy for approximately 2 weeks to allow hematological recovery. If the latter is achieved within the 2-week time frame, we proceed with cycle 2 without dose modifications. If myelosuppression persists beyond two weeks, we interrupt therapy until platelets > 50 × 10^9^/L and ANC > 0.5 × 10^9^/L with venetoclax administered for 21 days instead of 28 days during cycle 2. MRD assessment is also recommended once CR/CRi is achieved.

In the situation of persistent/residual AML post cycle 1, we proceed with cycle 2 of therapy noting that median time to first and best response is 1.2 and 2.1 months, respectively. If leukemia persists, without disease progression post cycle 2, it is reasonable to proceed with cycle 3 if no alternative therapeutic options. In our experience, one-third of patients experienced a delayed response after ≥3 cycles of therapy^20^.

Prolonged myelosuppression and treatment interruptions with subsequent cycles are a frequent occurrence^20^. Once morphological remission is achieved, we recommend delaying next cycle of therapy until platelets >50 × 10^9^/L and ANC > 0.5 × 10^9^/L. Step wise dose modifications are recommended with each cycle delay such as venetoclax administration for 21 days, followed by 14 and 7 days along with potential dose modifications of HMA; azacitidine 75 mg/m^2^ for 5 days or decitabine 20 mg/m^2^ for 3 days.

In frail elderly patients, we recommend a shortened course of venetoclax for 14 days starting cycle 1. Once CR/CRi/MLFS is achieved in the above instance, treatment cycles may be administered every 6–8 weeks with low-dose HMA along with an abbreviated course of venetoclax for 7 days.

## Discussion

Over the last 2 years, the approval of venetoclax-based therapy for upfront use in elderly or unfit AML patients has led to a paradigm shift in our management approach for AML, especially with recently reported phase-3 studies (VIALE-A, and VIALE-C) demonstrating a clear survival benefit with venetoclax combination therapies in treatment-naïve AML patients^15,18^. Remarkably, venetoclax-based therapy due to its minimal toxicity and high efficacy has enabled a subset of elderly AML patients to proceed to AHSCT. In regard to post-transplant outcomes following ventoclax-based therapy, a recent study highlights favorable transplant outcomes in 32 AML patients, of which 22 patients were transplanted in CR/CRi with 1 year overall survival of 77% and non-relapse mortality of 9.1%^52^. With respect to MDS, we await mature trial results documenting superior efficacy with venetoclax and it remains to be seen whether its addition to HMA confers a meaningful survival advantage.

An area of uncertainty with venetoclax-based therapy in AML involves the routine use and choice of antifungal prophylaxis. In clinical trials, azole antifungal prophylaxis was prohibited due to CYP3A4 inhibition since venetoclax is predominantly metabolized by CYP3A4. It remains unclear whether a lower dose of venetoclax administered with azoles compromises response outcomes. Fortunately, in study patients treated with venetoclax plus azacitidine, the incidence of grade 3/4 fungal infections was low at 8% which was attributed to use of echinocandins in 46% of patients^13^. In a large retrospective series of 119 AML patients treated at City of Hope with venetoclax plus HMA, 38% of patients received micafungin, 41% azole, and 21% no antifungal prophylaxis, with a low incidence of fungal infection at 5% in treatment naïve, vs 19% in relapsed AML, and 6% in responders vs 22% in non-responders^33^. In our practice, we prescribe azole antifungal prophylaxis, with posaconazole in relapsed or treatment naïve young AML patients while fluconazole is preferred in elderly patients.

Another unsettled issue is regarding the optimal timing and lack of uniformity of response assessment including MRD assessment^51,53^. Significant variations exist within terminology used to characterize responses other than CR which is defined as <5% marrow blasts, platelets >100 × 10^9^/L and ANC > 1.0 × 10^9^/L. On the contrary, the distinction between CRi (<5% marrow blasts, and either platelets >100 × 10^9^/L or ANC > 1.0 × 10^9^/L), CR with incomplete platelet recovery, (CRp), CR with partial hematologic recovery (CRh) (<5% blasts in the bone marrow, and partial recovery of peripheral blood counts (platelets > 50 × 10^9^/L and ANC > 0.5 × 10^9^/L), and MLFS, (marrow blast < 5% with >200 cells or marrow cellularity ≥10%, and no hematological recovery) is not well-defined. The time frame at which response assessment is performed is also variable based on goals of therapy with some physicians obtaining a bone marrow biopsy after completion of a minimum of two cycles of therapy. Similarly, MRD assessment is inconsistent in terms of timing and platform used; multicolor flow cytometry (MFC), digital droplet polymerase chain reaction (PCR), or emerging next generation sequencing technologies^54^. At our institution, multicolor flow cytometry based MRD assessment^55^ is routinely performed through the University of Washington, Seattle, for patients achieving CR/CRi particularly if AHSCT is being considered. The overall sensitivity of the assay is conservatively estimated as 0.1%. In addition, we have developed an in-house quantitative PCR assay for monitoring type A, B, and D *NPM1* mutation types with forthcoming assays for *CBFB/MYH11* and *RUNX1/RUNX1T1* fusions.

In terms of therapeutic decision-making for elderly or unfit AML patients with known *FLT3* or *IDH1/2* mutations, we are often faced with the choice of venetoclax-based therapy or targeted therapy with either *IDH* inhibitors (enasidenib/ivosidenib) or *FLT3* inhibitors (midostaurin/gilteritinib). Preliminary results from an ongoing phase Ib/II study on venetoclax plus ivosidenib with or without azacitidine in *IDH1* mutated AML and high-risk MDS appear promising^56^. A total of 19 patients were enrolled of which 17 were AML (9 relapsed, 5 treatment naïve and 3 with secondary AML following MDS with progression on HMA) and 2 patients with high-risk MDS with composite CR rate of 78% overall and 100% for treatment-naive patients. In addition, half of patients who achieved CR also were MRD negative. In a similar vein, preclinical studies confirm synergistic activity with BCL-2 and FLT3 inhibition^57^ with a phase 1b trial of venetoclax with gilteritinib in relapsed/refractory AML in progress. Of 15 relapsed/refractory AML patients, 10 of which were *FLT3* mutated (6 previously treated with FLT3 inhibitors), half achieved composite CR, and another 40% achieved MLFS^58^. Furthermore, a recently published Phase II study confirms the safety and efficacy of combination therapy with addition of FLT3 inhibitors to decitabine and venetoclax^16^. Together, these findings suggest that future therapies for AML are likely to incorporate targeted agents (*IDH/FLT3* inhibitors) into venetoclax-based regimens.
